# Factors predicting mortality in emergency abdominal surgery in the elderly

**DOI:** 10.1186/1749-7922-7-12

**Published:** 2012-05-11

**Authors:** Naoto Fukuda, Joji Wada, Michio Niki, Yasuyuki Sugiyama, Hiroyuki Mushiake

**Affiliations:** 1 Department of Surgery, Kawasaki Kyodo Hospital, Kawasaki 210-0833, Japan; 2 Department of Surgery, Teikyo University School of Medicine, University Hospital, Mizonokuchi, Kawasaki 213-8507, Japan

**Keywords:** Abdominal surgical emergency, Elderly, Mortality, Morbidity

## Abstract

**Objective:**

This study aimed to investigate clinical features of abdominal emergency surgery in elderly patients, and to determine factors predicting mortality in these patients.

**Methods:**

The study population included 94 patients aged 80 years or older who underwent emergency surgery for acute abdominal diseases between 2000 and 2010. Thirty-six patients (38.3%) were male and fifty-eight patients (61.7%) were female (mean age, 85.6 years). Main outcome measures included background of the patient’s physical condition (concomitant medical disease, and performance status), cause of disease, morbidity and mortality, and disease scoring system (APACHE II, and POSSUM). Prognostic factors affecting mortality of the patient were also evaluated by univariate analysis using Fisher’s exact test and Mann–Whitney U–test, and by multivariate analysis using multiple logistic regression analysis.

**Results:**

Of the 94 patients, 71 (75.5%) had a co-existing medical disease; most patients had hypertension (46.8%). The most frequent surgical indications were acute cholecystitis in 23 patients (24.5%), followed by intestinal obstruction in 18 patients (19.1%). Forty-one patients (43.6%) had complications during hospital stay; the most frequent were surgical site infection (SSI) in 21 patients (22.3%) and pneumonia in 12 patients (12.8%). Fifteen patients died (overall mortality, 16%) within 1 month after operation. The most common causes of death were sepsis related to pan-peritonitis in 5 patients (5.3%), and pneumonia in 4 patients (4.3%). Multiple logistic regression analysis showed that time from onset of symptoms to hospital admission and the POSSUM scoring system could be prognostic factors for mortality.

**Conclusions:**

Mortality in elderly patients who underwent emergency surgery for acute abdominal disease can be predicted using the disease scoring system (POSSUM) and on the basis of delay in hospital admission.

## Introduction

One of the most notable events being seen in recent years in people living in developed countries is an increased life span. In Japan, average life expectancy has gradually increased to 79.59 years among men and 86.44 years among women in 2009. Japan may be one of the most eminent countries where many people live to an advanced age because more than 50% of Japanese people survive over 80 years. As average life expectancy lengthens, the number of geriatric patients who need emergency abdominal surgery will increase. Compared with elective surgery, emergency abdominal surgery is associated with increased morbidity and mortality, especially in elderly patients [1-6]. Thus, elderly patients with abdominal surgical emergency may be at risk for severe and life-threatening conditions because of medical comorbidities, insufficient screening, unrecognized symptoms, and inadequate overall access to the health care system [7]. In this study, geriatric patients were limited to those aged 80 years or older because of increasing life expectancy in Japanese people. The aim of the present study was to report our experience with emergency abdominal surgery in the elderly patients and to identify risk factors that have an impact on mortality in these patients.

## Methods

Ninety-four patients ages 80 years or over who underwent emergency surgery for acute abdominal disease at our institutions between 2001 and 2010 were enrolled in this study. They included 36 men (38.3%) and 58 women (61.7%) ages 80–104 years (mean, 85.6 years). Of the 94 patients, 71 (75.5%) had co-existing medical diseases such as hypertension in 44 patients (46.8%), chronic heart disease in 17 (18.1%), chronic obstructive pulmonary disease (COPD) in 14 (14.9%), cerebrovascular disease and DM in 11 (11.7%) respectively, chronic renal failure in 6 (6.4%), and others in 12 (12.8%). Of the 71 patients with concomitant medical disease, 32 had 1 medical disease and 39 had 2 or more additional medical problems. The Eastern Cooperative Oncology Group (ECOG) performance status score [8], which reflects the daily living abilities of the patient was estimated for these patients and the results were as follows: 2 patients were with grade 0, 28 with grade 1, 48 with grade 2, 13 with grade 3, and 3 with grade 4 (Table 1). Of the 94 patients, 76 (80.9%) underwent emergency surgery within 48 hours after admission. The other18 patients (e.g., those with acute cholecystitis, intestinal obstruction due to adhesion) were first treated conservatively, and only when the conservative treatment failed did they undergo surgery.

**Table 1:** Of the 94 patients, 71 (75.5%) had co-existing medical diseases such as hypertension in 44 patients (46.8%), chronic heart disease in 17 (18.1%), chronic obstructive pulmonary disease (COPD) in 14 (14.9%), cerebrovascular disease and DM in 11 (11.7%) respectively, chronic renal failure in 6 (6.4%), and others in 12 (12.8%)

**Variables**	**n (%)**
Age 80-104 years (mean: 85.6)	
Gender	
Male	36 (38.3%)
Female	58 (61.7%)
Co-existing medical disease	
Hypertension	44 (46.8%)
Chronic heart disease	17 (18.1%)
COPD	14 (14.9%)
Cerebrovascular disease	11 (11.7%)
DM	11 (11.7%)
Chronic renal failure	6 (6.4%)
Others	12 (12.8%)
Performance status (ECOG)	
Grade 0	2 (2.1%)
Grade 1	28 (29.8%)
Grade 2	48 (51.1%)
Grade 3	13 (13.8%)
Grade 4	3 (3.2%)

The ECOG performance status score were as follows: 2 patients were with grade 0, 28 with grade 1, 48 with grade 2, 13 with grade 3, and 3 with grade 4.

Data regarding the patient’s clinical features, surgical outcomes including morbidity and mortality, and follow-up information were obtained from a clinical database. We evaluated clinical factors that could be associated with mortality in abdominal emergency surgery in elderly patients. These parameters included age, gender, background of the patient’s physical condition (concomitant medical disease, and ECOG performance status [8]), time from onset of symptom to hospital admission, and disease severity scoring system (APACHE II [9], and POSSUM [10]). Physiological Score (PS) and Operative Severity Score (OSS) in POSSUM scoring system [10] as well as APACHE II score [9] were analyzed as parameters of the disease scoring system. For statistical analysis, the patients were grouped into 2 categories with respect to age [≤85 years or >85 years (mean value)], comorbidity (negative or positive), ECOG performance status score (Grade0 or 1 vs. Grade2 or 3 or 4), and time from onset of symptom to hospital admission (<24 h or ≥24 h). Post-operative morbidity and mortality were defined as operation-related complications or death that occurred within 30 days after the operation. Univariate comparison between the groups were performed using the Fisher’s exact test and Mann–Whitney U − test.

Covariates that remained significant through univariate analysis were selected for multivariate analysis. Multivariate analysis was performed using the multiple logistic regression analysis. The results were evaluated at a confidence interval of 95% and significance was set at p < 0.05.

This study was carried out in compliance with the Helsinki Declaration. Written informed consent was obtained from the patient for publication of this report and any accompanying images.

## Results

### Causes of acute abdomen

**Figure 1:**
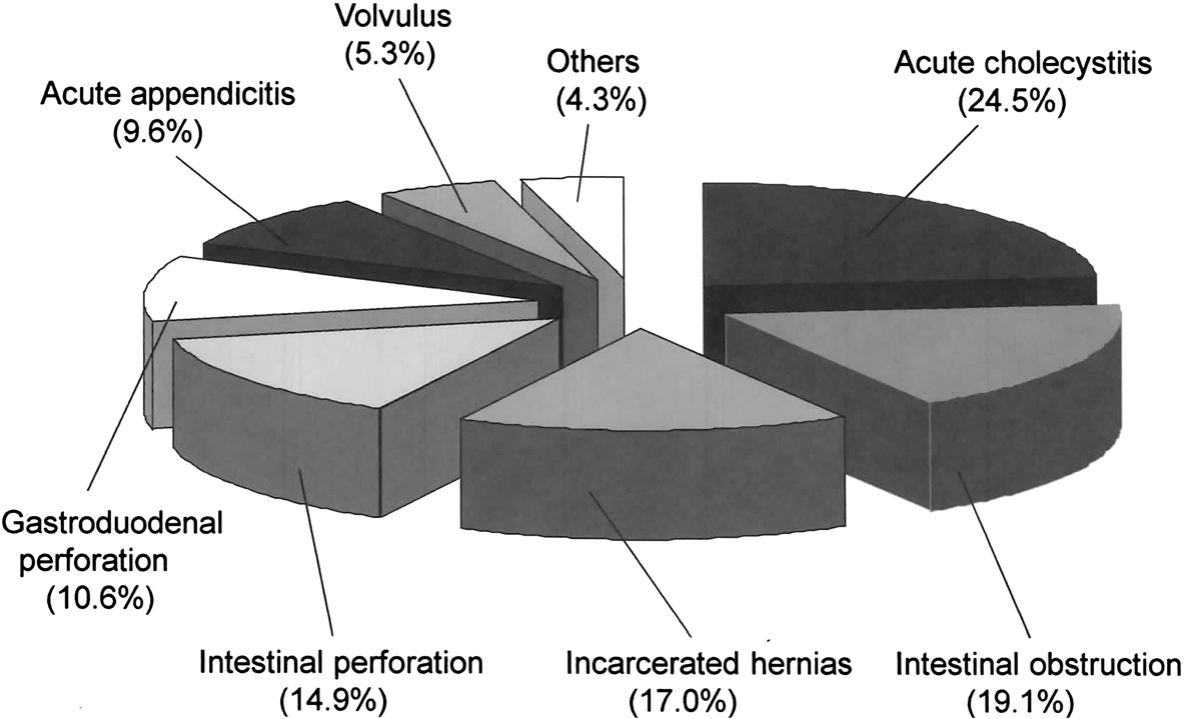
**The most frequent surgical indications were acute cholecystitis in 23 patients (24.5%), followed by intestinal obstruction in 18 patients (19.1%).** There were also 16 cases (17.0%) of incarcerated hernias, 14 cases (14.9%) of intestinal perforation, 10 cases (10.6%) of gastro-duodenal perforation, 9 cases (9.6%) of acute appendicitis, 5 cases (5.3%) of volvulus, and 4 cases (4.3%) of other acute abdominal disease.

The most frequent surgical indications were acute cholecystitis in 23 patients (24.5%), followed by intestinal obstruction in 18 patients (19.1%). There were also 16 cases (17.0%) of incarcerated hernias, 14 cases (14.9%) of intestinal perforation, 10 cases (10.6%) of gastro-duodenal perforation, 9 cases (9.6%) of acute appendicitis, 5 cases (5.3%) of volvulus, and 4 cases (4.3%) of other acute abdominal disease (Figure 1).

Intestinal obstruction was most often caused by adhesion (n = 14); two patients needed intestinal resection due to intestinal necrosis and perforation. Other etiologies of intestinal obstruction were colonic malignancy (n = 2), internal hernia (n = 1), and gallstone ileus (n = 1). Incarcerated hernias consisted of 9 cases of femoral hernias, 4 cases of inguinal hernias, 2 cases of obturator hernias, and 1 case of incisional hernia. Among the cases of intestinal perforation, 5 cases were small intestinal perforations and 9 cases were large intestinal perforations. The most common cause of intestinal perforation was incarcerated hernia (n = 4), followed by colon diverticulitis (n = 3). Gastro − duodenal perforations were found in 5 cases of perforated duodenal ulcer, 3 cases of perforated gastric ulcer, 1 case of duodenal perforation due to gallbladder cancer invasion, and 1 case of iatrogenic gastric perforation caused by guide-wire of a long tube using for intestinal obstruction.

### Treatment

All patients were treated surgically. Seventy-six patients (80.9%) underwent emergency surgery within 48 hours after admission; the other 18 patients were first treated conservatively and then operated on more than 48 hours after admission. The most common operation was intestinal resection (n = 30), followed by cholecystectomy (n = 24), repair of intestinal adhesion (n = 15), and hernia repair (n = 14). Of the 30 patients treated with intestinal resection, large bowel resection was applied to 17 patients, and small bowel resection to 13 patients. Cholecystectomy was performed laparoscopically in 3 patients, and using laparotomy in 21 patients. There were only 3 cases of palliative surgery; 1 ileostomy for transverse colon perforation, 1 peritoneal lavage for acute pancreatitis, and 1 gastroduodenostomy for advanced gallbladder cancer. Twenty-three patients (24.5%) were followed in the intensive care unit after surgery. Of these, 20 patients needed mechanical ventilation for respiratory support.

### Morbidity and mortality

Forty-one patients (43.6%) had post − operative morbidity. The most frequent complication was surgical site infection (SSI), which occurred in 21 patients (22.3%), followed by pneumonia in 12 patients (12.8%). Sepsis occurred in 5 patients (5.3%), DIC in 5 patients (5.3%), and ARDS, acute renal failure, anastomosis leakage, and urinary tract infection occurred in 2 patients (2.1%) respectively. Of the 12 cases of pneumonia, more than half (8 patients) were aspiration pneumonias. Fifteen patients (16.0%) died within 1 month after their operation. The most common causes of death were sepsis related to pan-peritonitis in 5 patients (5.3%), and pneumonia in 4 patients (4.3%). The other etiologies of mortality consisted of 2 cases of cancer, 1 multiple organ failure, 1 intraperitoneal bleeding due to DIC, 1 renal failure, and 1 suffocation. These complications are listed in Table 2.

**Table 2:** Forty-one patients (43.6%) had post-operative morbidity

	**Patient (n = 94)**	**%**
Morbidity	41	43.6
SSI	21	22.3
Pneumonia	12	12.8
DIC	5	5.3
Sepsis	5	5.3
ARDS	2	2.1
Acute renal failure	2	2.1
Anastomosis leakage	2	2.1
Urinary tract infection	2	2.1
Mortality	15	16.0
Sepsis	5	5.3
Pneumonia	4	4.3
Cancer	2	2.1
Multiple organ failure	1	1.1
Intraperitoneal bleeding	1	1.1
Renal failure	1	1.1
Suffocation	1	1.1

The most frequent complication was surgical site infection (SSI), which occurred in 21 patients (22.3%), followed by pneumonia in 12 patients (12.8%). Fifteen patients (16.0%) died within 1 month after their operation. The most common causes of death were sepsis related to pan-peritonitis in 5 patients (5.3%), and pneumonia in 4 patients (4.3%).

### Clinical factors affecting mortality

Clinical factors that might affect the mortality of elderly patients treated with emergency abdominal surgery were evaluated. Delay in hospital admission (more than 24 hours after onset of symptom), APACHE II score, and POSSUM score (PS, OSS) were identified as prognostic factors of these patients on univariate analysis (Table 3). Additionally, multivariate analysis using multiple logistic regression analysis demonstrated that delay in hospital admission (*p* = 0.0076) and POSSUM score (PS) (*p* = 0.0301) were effective prognostic factors of elderly patients who underwent emergency abdominal surgery (Table 4).

**Table 3:** Delay in hospital admission (more than 24 hours after onset of symptom), APACHE II score, and POSSUM score (PS, OSS) were identified as prognostic factors of these patients on univariate analysis

	**Alive (n = 79)**	**Dead (n = 15)**	***P***
Age (mean: 85.6)			
≤85	41	10	
>85	38	5	0.2219
Gender			
Male	27	9	
Female	52	6	0.0567
Comorbidity			
negative	20	3	
positive	59	12	0.4715
PS(ECOG)			
Grade 0,1	28	2	
Grade 2, 3, and 4	51	13	0.0786
Time from onset of symptoms to hospital admission (hour)			
<24	51	4	
≥24	28	11	0.0074** (Fisher’s exact test)
APACHE II (mean)	11.9	18.5	0.0002
POSSUM			
PS (mean)	30.1	38.6	0.0001**
OSS (mean)	13.9	17.2	0.0408* (Mann-Whitney U-test)

**Table 4:** Multivariate analysis using multiple logistic regression analysis demonstrated that delay in hospital admission (p=0.0076) and POSSUM score (PS) (p=0.0301) were effective prognostic factors of elderly patients who underwent emergency abdominal surgery

		**Odds ratio**	**95% CI**	***p***
Time from onset to hospital admission (>24 hr vs. 24 hr)		9.6039	1.8226-50.6079	0.0076**
APACHE II		1.1291	0.9223-1.3822	0.2395
POSSUM	PS	1.2013	1.0178-1.4178	0.0301*
	OSS	1.0202	0.8468-1.2292	0.8331

## Discussion

As the increase of life expectancy has been observed in developed countries, especially in Japan, the number of geriatric patients with acute abdominal disease requiring emergency surgical treatment has increased in recent decades. Because physiological reserve is significantly diminished in the elderly, cardiovascular, pulmonary, endocrine, and renal comorbidities are more common in elderly patients. Previous studies [1,6,11-13] have shown that the incidence of comorbidity in the elderly with acute abdominal disease requiring emergency operation was more than 50%, ranging from 58 to 81.5%. Our study also revealed that the rate of having co-existing medical disease in the aged patient was 75.5%, and hypertension (46.8%) was the most common comorbidity, followed by chronic heart disease (18.1%), and COPD (14.9%). The presence of underlying chronic conditions may have an adverse effect on the prognosis in patients undergoing emergency surgery and may be responsible for the increased perioperative risk, and consequently, mortality. Ozkan [13] reported that all patients who died postoperatively had at least 1 comorbid condition, whereas comorbid conditions existed in 66.3% of the surviving patients in the study of emergency abdominal surgery in geriatric patients. On the other hand, Rubinfeld [14] showed that none of the comorbidities accurately predicted mortality in the patients aged 80 years and older who received an emergency major abdominal operation. Our study also revealed that comorbidity was not a significant prognostic factor for elderly patients with abdominal surgical emergency on univariate analysis *(p* = 0.4715).

According to the results, underlying medical disease may not affect the mortality of the elderly patient with acute abdominal disease requiring emergency operation, because appropriate management of medical comorbidities due to development of medical technology in recent decades may improve the prognosis of the elderly patient with underlying medical problems.

In the current study, the complication rate was as high as 43.6%, which is similar to those reported previously [1,4,6,15]. Surgical site infection (SSI) was the most frequent complication and occurred in 21 patients (22.3%), followed by pneumonia in 12 patients (12.8%). Arenal [6] reported that 48% of the patients had morbidity, the majority of which was wound infection (16.3%), followed by respiratory complications (11.4%) and cardiac complications (8.9%) in a study of 710 patients ages 70 years or older who underwent emergency surgery for intra-abdominal disorders. Thus, wound infection which is a local morbidity may be the most frequent complication after emergency operation for acute abdominal disease in elderly patient.

Among the systemic morbidities, cardio-pulmonary complications are more common in the elderly patients compared to younger patients because cardio − pulmonary function declines with aging. Our study also revealed that 12.8% of the patients had post − operative pneumonias, in which more than half of the cases were aspiration pneumonias. As swallowing ability is diminished in the elderly, especially those aged 80 years or more, we must pay more attention to aspiration pneumonia in the elderly patient after surgical treatment for acute abdominal disease.

Despite the relatively high incidence of morbidity (43.6%), the mortality of our patients was 16.0%. This result is similar or better than that of previously published reports, which ranged from 11 to 34% [4-6,13,14,16].

The most common causes of death in elderly patients in our study were sepsis related to pan-peritonitis (5.3%) and pneumonia (4.3%); these findings were similar to those of previous reports [13] in which post-operative pneumonia, cardiac complications and sepsis accounted for a large proportion of deaths in elderly patients. Cancer was reported to be the most common reason for death in elderly patients with abdominal emergency surgery in another study [4]. The different conclusions in that study might be explained by different patient populations, especially the number and percentage of patients with oncological emergency.

Many factors have been reported to be responsible for surgical mortality during acute abdomen in elderly patients. The most common factor was ASA score, which consists of 6 categories to evaluate the degree of a patient’s sickness or physical status, and that was reported as an independent prognostic factor in 3 previous studies [6,13,14]. ASA score is ordinarily used to assess the patient’s physical status before surgery by an anesthesiologist, whereas it is not commonly used by surgeons. The POSSUM scoring system developed by Copeland [10] in 1991 has since been applied to a number of surgical groups as surgical culture moves more towards outcome measures and providing the patient with as much information as possible to make fully informed decisions. The POSSUM scoring system has 2 main components: Physiological Score (PS) and Operative Severity Score (OSS). PS is based on 12 physiological parameters to evaluate a patient’s physiological status before surgery, whereas OSS consists of 6 operative parameters accounting for the severity of the procedure. Since the ASA score is too simplistic and highly subjective compared to the APACHE II or POSSUM scoring system, we chose APACHE II and POSSUM (PS, OSS) as disease scoring systems instead of the ASA score in the study of prognostic factors for elderly patients who undergo emergency abdominal surgery. Consequently, the POSSUM score (PS) was identified as an effective prognostic factor in elderly patients who underwent emergency abdominal surgery on multivariate analysis. Since the PS in the POSSUM scoring system is objective and reflects the patient’s overall condition, including his age, vital signs, blood chemistry, mental status and heart condition, it may be more effective than the ASA score for evaluating the prognosis of elderly patients with abdominal surgical emergencies. Another effective prognostic factor defined in the present study was delay in hospital admission (more than 24 hours after onset of symptoms).

The prognosis of the patient who was admitted more than 24 hours after onset of symptoms was significantly worsened than that of the patient who admitted within 24 hours on multivariate analysis (p = 0.0076). The difficulty in obtaining an accurate history and the mild character of symptoms due to decline in organ and tissue function in the aged patient have an effect on the findings, resulting in delayed diagnosis and a more complicated peri-operative period. Socioeconomic factors may also play a role because the elderly patient may not have adequate access to the health care system, which might be one of the reasons for delay in hospital admission. Because elderly patients with acute abdominal disease tend to have delayed diagnoses and surgical treatments, rapid access to the hospital, adequate diagnostic measures and decision-making should be required to prevent postoperative complications and to improve the prognosis.

## Conclusions

POSSUM scoring system (PS) and delay in hospital admission may be prognostic factors for mortality in elderly patients who underwent emergency surgery for acute abdominal disease.

## Competing interests

The authors declare that they have no competing interests.

## Authors’ contributions

Study Design/Data Collection/Analysis/Interpretation: FN. Manuscript Drafting: HM. Critical Review: YS. All authors read and approved the final manuscript.
